# A new synonym of *Polygonatum* in China, based on morphological and molecular evidence

**DOI:** 10.3897/phytokeys.175.63383

**Published:** 2021-04-12

**Authors:** Maoqin Xia, Ying Liu, Jingjing Liu, Donghong Chen, Yan Shi, Zhicong Bai, Yu Xiao, Chen Peng, Jinping Si, Pan Li, Yingxiong Qiu

**Affiliations:** 1 Laboratory of Systematic & Evolutionary Botany and Biodiversity, College of Life Sciences, Zhejiang University, Hangzhou 310058, China Zhejiang University Hangzhou China; 2 State Key Laboratory of Subtropical Silviculture, Zhejiang A&F University, Lin’an, Hangzhou 311300, China Zhejiang A&F University Hangzhou China

**Keywords:** *
Polygonatum hunanense
*, Polygonatum kingianum var. grandifolium, phylogeny, plastome

## Abstract

PolygonatumkingianumCollett et Hemsl.var.grandifolium D.M. Liu & W.Z. Zeng (1981), which sprouts twice a year, once in spring and once in autumn, differs from *Polygonatumkingianum* in leaves, bracts, perianth and filaments. Morphological comparison and molecular phylogeny indicate that it is identical to the newly-published *Polygonatumhunanense* H.H. Liu & B.Z. Wang (2021). Hence, we propose that P.kingianumvar.grandifolium should be recognised as a new synonym of *P.hunanense*. In addition, phylogenetic analyses confirmed that *P.hunanense* is sister to Polygonatumsect.Polygonatum, rather than *P.kingianum* of Polygonatumsect.Verticillata.

## Introduction

The genus *Polygonatum* Mill. (Asparagaceae, tribe Polygonateae), commonly known as ‘Solomon’s Seal’, contains more than 60 species widespread in the Northern Hemisphere, with Himalayas to southwest China and north-eastern Asia as diversification centres (Tamura et al. 1997a; Jeffrey 1980, 1982; Wang et al. 2016). Species in *Polygonatum* are perennial herbs with rhizome, stems erect, arching or sometimes scandent, leaves alternate, opposite or whorled, inflorescences an umbel, corymb or raceme (Chen and Tamura 2000). Rhizomes of some species, like *Polygonatumsibiricum* Redouté, *Polygonatumcyrtonema* Hua and *Polygonatumkingianum* Collett et Hemsl., are widely used in traditional Chinese medicine. Tamura et al. separated *Heteropolygonatum* M.N. Tamura et Ogisu from *Polygonatum* (Tamura et al. 1997a) and found the topological difference of *Heteropolygonatum* and its relative taxa: (1) (*Heteropolygonatum* + *Disporopsis*) + *Polygonatum* and (2) (*Heteropolygonatum* + *Polygonatum*) + *Disporopsis* (Tamura et al. 1997b). Later, the former case was supported by Meng et al. (2014) and Wang et al. (2016), whereas the latter one was supported by Floden and Schilling (2018) and Zhao et al. (2019). The genus *Polygonatum* was divided into three sections, based on four chloroplast molecular markers, leaf arrangement and basic chromosome number: (1) Polygonatumsect.Polygonatum, which is characterised by alternate leaves and basic chromosome number x = 9–11; (2) Polygonatumsect.Sibirica only includes *Polygonatumsibiricum* with whorled leaves and x = 12; and (3) Polygonatumsect.Verticillata shows variable phyllotaxy and x = 13–15 (Meng et al. 2014). This is widely accepted and confirmed by multiple molecular phylogenetic studies (Wang et al. 2016; Floden and Schilling 2018; Zhao et al. 2019). However, due to hybridisation and polyploidisation in this genus, classification of some species with large morphological variations and wide distribution range remains controversial (Tamura 1990; Tamura 2008; Floden 2015; Floden and Schilling 2018; Zhao et al. 2019).

**Figure 1.**
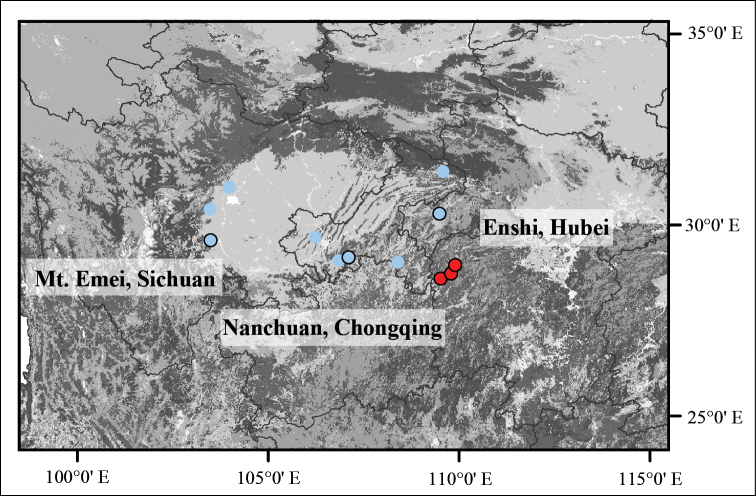
Distribution map of *Polygonatumhunanense* (red dots with black circle, based on Liu et al. 2021) and Polygonatumkingianumvar.grandifolium (blue dots with black circle: three populations investigated in this study; blue dots: previous specimen records).

**Figure 2.**
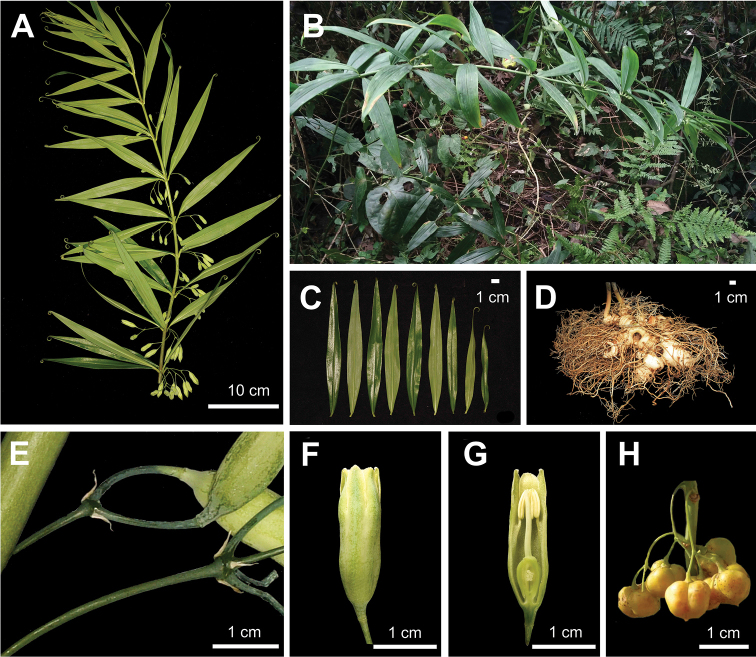
Polygonatumkingianumvar.grandifolium**A** stem **B** plant habit **C** leaves **D** rhizome **E** bracts **F** flower **G** longitudinal section of flower **H** fruit.

**Figure 3.**
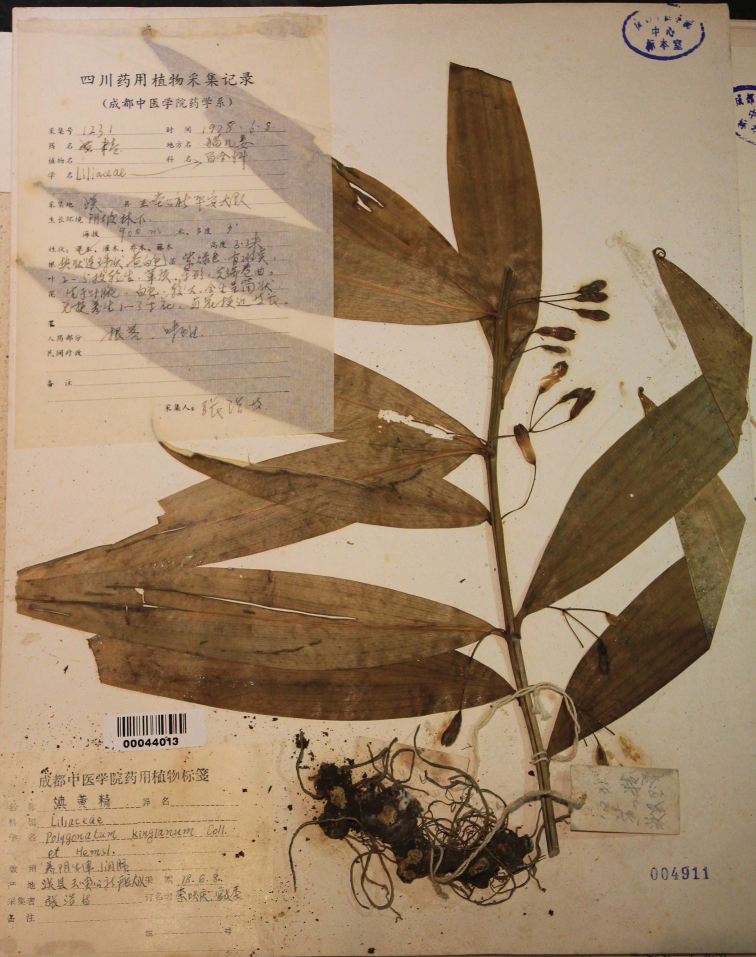
Lectotype of Polygonatumkingianumvar.grandifolium, *Hao Zhang 1231* (CDCM 00044013).

**Figure 4.**
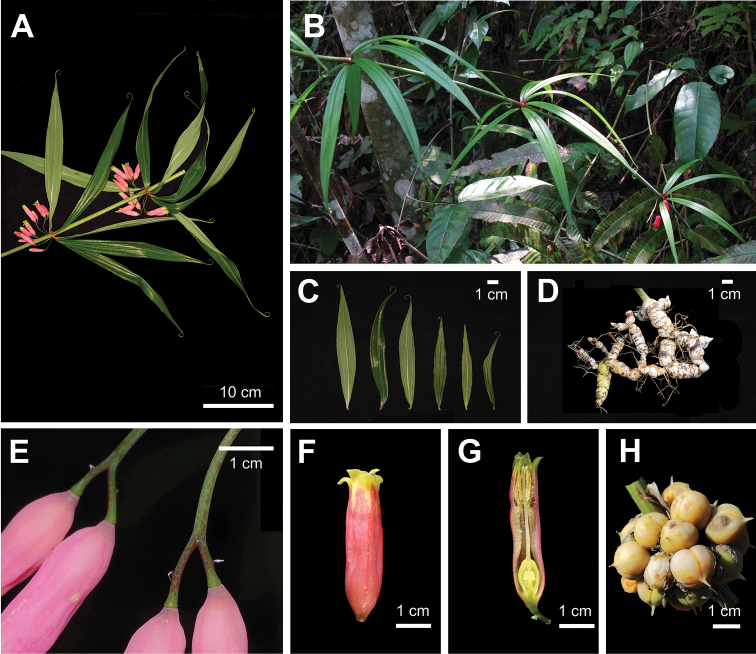
*Polygonatumkingianum***A** stem **B** plant habit **C** leaves **D** rhizome **E** bracts **F** flower **G** longitudinal section of flower **H** fruit.

During fieldwork in the last few years, we found several populations of a unique *Polygonatum* species in Sichuan Province, Chongqing Municipality and Hubei Province of China (Figure 1). The plants are 1–3 m high with 3–5 whorled leaves per round, yellowish-white or greenish-white flowers and yellow or orange berries (Figure 2). It sprouts twice a year, once in spring (March to April) and once in autumn (September), whereas other species sprout only once in spring. It is likely belonging to the section Verticillata, according to the phenotype. After carefully checking the protologue and type specimens (Figure 3), we found that our collections matched the description of PolygonatumkingianumCollett et Hemsl.var.grandifolium D.M. Liu et W.Z. Zeng, which was published in Flora Sichuanica and is differing from other *P.kingianum* varieties by having broader leaves ((1.5–) 2.4–5 cm wide) with green leaf base (vs. 0.2–1.0 (–1.5) cm wide, leaf base red), 2-5 mm long bracts at base of pedicel (vs. 1-2 mm long, on pedicel), yellowish- or greenish-white perianth (vs. pink, red or white) (Figures 2, 4; Xu 1981). In addition, it is also similar to the newly-published *Polygonatumhunanense* H.H. Liu & B.Z. Wang from Hunan, China (Liu et al. 2021). In this study, molecular phylogenetic analyses were performed to reveal the phylogenetic relationships amongst P.kingianumvar.grandifolium, *P.hunanense* and *P.kingianum*.

## Materials and methods

### Morphologic observation

Morphological characters of the living individuals from Mt. Emei, Sichuan Province, China were observed. In addition, 16 herbarium specimens of Polygonatumkingianumvar.grandifolium in IMC, CDCM and CDBI were examined. Subsequently, morphological comparisons were conducted with the living individuals, specimens and descriptions of *Polygonatumhunanense* and *Polygonatumkingianum* from flora and previous research (Jeffrey 1980; Xu 1981; Chen and Tamura 2000; Liu et al. 2021).

### Sequencing, plastome assembly and annotation

In order to determine the phylogenetic status of the taxon, we sequenced three samples from Nanchuan, Chongqing Municipality, Enshi, Hubei Province and Mt. Emei, Sichuan Province, respectively (Figure 1), as well as two *Polygonatumkingianum*, one *Polygonatumsibiricum* and one *Polygonatumzanlanscianense* (Table 1). Representative voucher specimens are currently deposited at the Herbarium of Zhejiang University (HZU). Genomic DNAs were extracted from silica-gel dried leaves using DNA Plantzol Reagent (Invitrogen), following the manufacturer’s instructions. The libraries were prepared and sequenced using paired-end 150 bp at Beijing Genomics Institute (BGI, Shenzhen, China) on a BGISEQ-500 sequencing platform. Approximately 3G raw data were generated for each sample. Raw data were trimmed by removing adapters and low-quality reads and then a de novo approach was applied to assemble plastomes using the NOVOPlasty v.3.8.3 (Dierckxsens et al. 2017) with K-mer = 39. The plastome and *rbc*L gene sequences of *Polygonatumstenophyllum* Maxim. (KX822773) were adopted as reference and seed sequence, respectively. DOGMA (Wyman et al. 2004) was used for plastome annotation with manually checking the start/stop codons in Geneious 10.2.3 (http://www.geneious.com). In addition, plastome data of *Polygonatum* and outgroups (*Heteropolygonatum* and *Disporopsis* Hance) from Floden & Schilling (2018) were used for phylogenetic analyses (Table 1). To study the phylogenetic relationship between P.kingianumvar.grandifolium and *P.hunanense*, *rbc*L, *trn*K, *psb*A-*trn*H and *trn*C-*pet*N, sequences from Liu et al. (2021) were downloaded for further phylogenetic analyses.

**Table 1.** GenBank accessions of plastomes involved in this study. Samples in bold were newly sequenced in this study.

Species name	GenBank number	Length
*Disporopsisjinfushanensis* Z.Y. Liu	MH891733	155,188
*Heteropolygonatumaltelobatum* (Hayata) Y.H. Tseng, H.Y. Tzeng et C.T. Chao	MH891734	155,534
*Heteropolygonatumalternicirrhosum* (Hand.-Mazz.) Floden	MH891737	155,510
*Heteropolygonatummarmoratum* (H. Lév.) Floden	MH891735	155,447
*Heteropolygonatumpendulum* (Z.G. Liu et X.H. Hu) M.N. Tamura et Ogisu	MH891736	155,436
*Polygonatumacuminatifolium* 2 Komarov	MH891751	155,304
*Polygonatumannamense* Floden	MH891738	155,277
*Polygonatumarisanense* Hayata	MH891752	155,340
*Polygonatumbiflorum* (Walter) Elliott	MH891756	155,470
*Polygonatumcathcartii* Baker	MH891745	155,970
*Polygonatumgovanianum* Royle	MH891755	155,089
**Polygonatumkingianumvar.grandifolium 1**	**MW373518**	**155,609**
**Polygonatumkingianumvar.grandifolium 2**	**MW373529**	**155,632**
**Polygonatumkingianumvar.grandifolium 3**	**MW373520**	**155,609**
*Polygonatumhuanum* H. Lév.	MH891743	155,545
***Polygonatumkingianum* 1 Collett et Hemsley**	**MW373516**	**155,810**
***Polygonatumkingianum* 2**	**MW373517**	**155,824**
*Polygonatummengtzense* 1 F.T. Wang et Tang	MH891740	155,498
*Polygonatummengtzense* 2	MH891741	155,492
*Polygonatumoppositifolium* (Wall.) Royle	MH891746	155,760
*Polygonatumorientale* Desf.	MH891753	155,386
*Polygonatumpunctatum* Royle ex Kunth	MH891739	155,333
***Polygonatumsibiricum* 1 Redouté**	**MW373521**	**155,549**
*Polygonatumstenophyllum* Maxim.	KX822773	156,028
*Polygonatumstewartianum* Diels	MH891749	155,867
*Polygonatumtessellatum* F.T. Wang et Tang	MH891747	155,724
*Polygonatumuncinatum* Diels	MH891744	155,694
*Polygonatumurceolatum* (J.M.H. Shaw) Floden	MH891742	155,504
*Polygonatumverticillatum* 1 (L.) Allioni	MH891748	155,878
*Polygonatumverticillatum* 2	MH891750	155,502
*Polygonatumyunnanense* H. Lév.	MH891754	155,363
***Polygonatumzanlanscianense* 1 Pampanini**	**MW373522**	**155,911**

### Phylogeny of *Polygonatum*

The sequence of 78 protein coding genes (CDS) shared by all plastomes were aligned using MAFFT v.7 (Katoh and Standley 2013) in Geneious 10.2.3. The *rbc*L, *trn*K, *psb*A-*trn*H and *trn*C-*pet*N sequences from Liu et al. (2021) and those from the seven newly-reported plastomes were aligned using MUSCLE in Geneious 10.2.3. DNASP6 was used to do statistics of site information (Rozas et al. 2017). The phylogenetic trees were constructed using both Maximum Likelihood (ML) and Bayesian Inference (BI) methods, implemented on CIPRES Science Gateway website (https://www.phylo.org, Miller et al. 2010) with the best-fit model of DNA substitution estimated by jModelTest v.2.1.4 (Darriba et al. 2012). ML analysis was conducted using RAxML-HPC BlackBox 8.2.12 with 1000 bootstrap replicates (Stamatakis 2014). Bayesian analysis was constructed using MrBayes XSEDE 3.2.7 with two independent Markov Chain Monte Carlo chains for 10,000,000 generations and sampling every 1000 generations (Ronquist and Huelsenbeck 2003). The first 25% of calculated trees were discarded as burn-in and the remaining trees were used to construct a consensus tree to estimate the posterior probability (PP).

## Results and discussion

Morphological comparisons showed that P.kingianumvar.grandifolium is almost the same as *P.hunanense*, except that the latter has narrower leaves (Table 2). However, they both differ from other *P.kingianum* varieties in leaves, bracts, perianth and filaments (Table 2). In addition, we have observed stout and no thickening filaments in P.kingianumvar.grandifolium (Figure 2G and Suppl. material 1: Figure S1) and slender filaments in *P.kingianum* (Figure 4G and Suppl. material 1: Figure S1). Tamura has reported that species of sect. Verticillata has slender filaments, whereas sect. Polygonatum has stout filaments and filaments of the ser. Bracteata, ser. Polygonatum and ser. Inflata are thickening in the upper part, thickening in the middle or without thickening and thickening in the lower part, respectively (Tamura 1991, 1993; Tamura et al. 1997b).

**Table 2.** Comparison of morphological characters amongst *P.hunanenses*, P.kingianumvar.grandifolium and *P.kingianum* varieties. The dashed line indicates the characters are the same as the original variety.

Characters	* P. hunanense *	P. kingianum var. grandifolium	* P. kingianum *			
			var. kingianum	var. cavaleriei	var. ericoideum	var. uncinatum
Rhizomes	moniliform or ginger-like, 1–4 cm thick	moniliform or ginger-like, 1–3.5 cm thick	subterete or submoniliform, 1–3 cm thick	–	–	–
Stem	1–3.5 m, apex subscandent	1–3 m, apex subscandent	1–3 m, apex subscandent	–	–	ca. 60 cm
Phyllotaxy	whorled, 3–6 (–10) per round	whorled, 3–5 per round	whorled, 3–10 per round	–	–	whorled, 4–5 per round
Leaf	5–20 (–27) cm × 0.5–2.5 (–3.2) cm, linear to lanceolate, apex strongly cirrose or curved	13–27 cm × (1.5–) 2.4–5 cm, linear to lanceolate, apex strongly cirrose or curved	6–16 (–20) cm × 0.2–1.0 (–1.5) cm, linear to lanceolate, membranous, apex cirrose	linear to lanceolate, somewhat coriaceous	narrow linear	short lanceolate, somewhat coriaceous, 5–6 cm × 1–1.4 cm
Inflorescence	(1–) 2–5	(1–) 2–5	(1–) 2–4 (–6)	1–2	2–4	1–2
Peduncle	1.7–3.5 cm	1–4 cm	1–2 cm	stout, strongly deflexed	2–3 cm	decurved
Pedicel	0.7–1.8 cm	0.4–1.7 cm	0.5–1.5 cm			–
Bract	subulate to lanceolate, 3–4 mm, at base of pedicel	linear, 2–5 mm, at base of pedicel	linear, 1–2 mm, on proximal part of pedicel			
	white or pale yellowish-green	yellow or greenish-white	pink, red	white, tinged purple	white	white
Perianth	cylindrical campanulate	cylindrical	cylindrical–campanulate	–	–	–
	17–22 mm	15–27 mm	18–25 mm	–	–	10–13 mm
Lobes	5–6.5 mm	4–6.5 mm	3–5 mm	–	–	–
Filament	2–3 mm, flat	2.5–3.5 mm, stout and no thickening	1.7–5 mm, slender	–	–	–
Anthers	ca. 5 mm	2.5–5.5 mm	4–6 mm	–	–	–
Ovary	5–7 mm	ca. 4 mm	4–6 mm	–	–	–
Style	ca. 9–12 mm	ca. 8–14 mm	(8–) 10–14 mm	–	–	–
Fruits	berries pale yellowish-green or orange, 1–1.8 cm	berries yellow or orange with black spots, 1.4–1.8 cm	berries yellow, orange or red, 1–1.5 cm	–	–	–
Seeds	3–12	5–15	7–12	–	–	–
Distribution	Hunan, China	Southwest China	Southwest China, Myanmar, Thailand, Vietnam	China: Sichuan, Yunnan	China: Yunnan	China: Yunnan
Altitude	200–700 m	600–1200 m	700–3600 m	–	–	–

**Figure 5.**
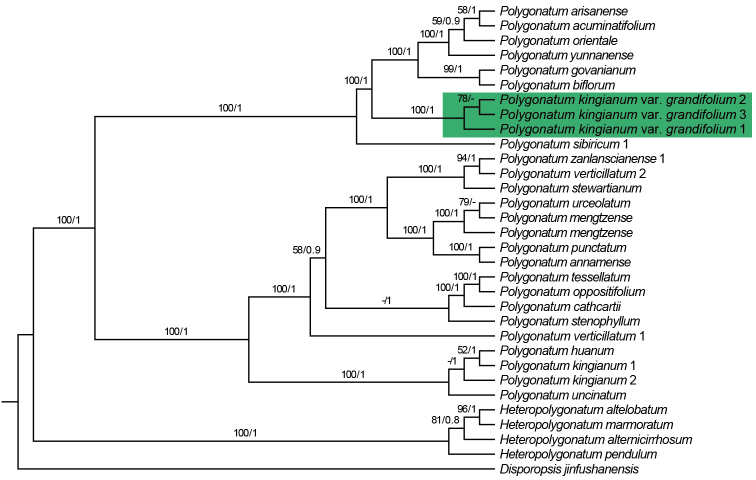
Phylogeny of *Polygonatum*, based on 78 protein coding genes (CDS) of plastome. Numbers above branches are Maximum Likelihood bootstrap values (BS)/Bayesian posterior probability (PP). The dash indicates support values of less than 50%. The phylogenetic position of Polygonatumkingianumvar.grandifolium is highlighted in green.

**Figure 6.**
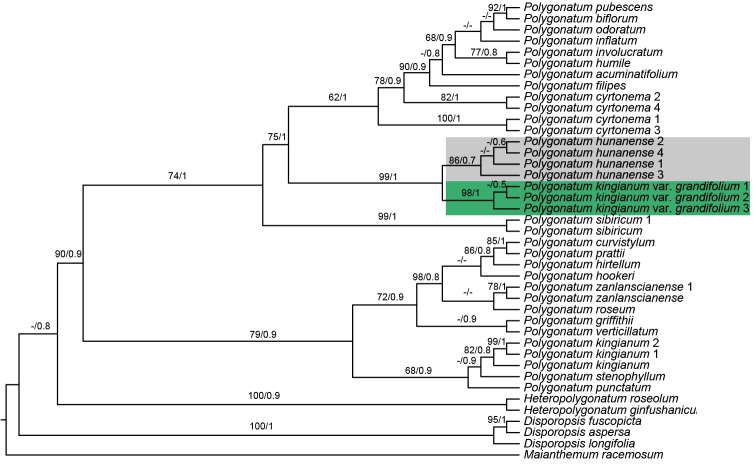
Phylogeny of *Polygonatum*, based on *rbc*L, *trn*K, *psb*A-*trn*H and *trn*C-*pet*N sequences. Numbers above branches are Maximum Likelihood bootstrap values (BS)/Bayesian posterior probability (PP). The dash indicates support values of less than 50%. The phylogenetic position of Polygonatumkingianumvar.grandifolium and *P.hunanense* are highlighted in green and grey, respectively.

The length of seven plastomes ranged from 155,549 bp to 155,911 bp, the accession numbers being MW373516−MW373522 (Table 1). They displayed the typical quadripartite structure with 132 genes in the same order, of which 112 were unique genes including 78 protein-coding genes, 30 tRNA genes and 4 rRNA genes. The alignment CDS matrix has 66,589 characters in length, in which 1827 are variable (polymorphic) sites and 512 are parsimony-informative sites. In addition, the alignment matrix of four plastid fragments has 4,652 characters in length, of which 85 are variable (polymorphic) sites and 58 are parsimony-informative sites. The phylogenetic tree of 78 CDS supports a robust monophyletic clade of three samples of P.kingianumvar.grandifolium (BS = 100, PP = 1; Figure 5). However, it was not closely related to *P.kingianum* of section Verticillata, but was sister to the section Polygonatum (BS = 100, PP = 1; Figure 5). Additionally, the phylogeny, based on four plastid fragments, supported the monophyly of *P.hunanense* and P.kingianumvar.grandifolium (BS = 99, PP = 1; Figure 6), which suggested they should be conspecific.

Therefore, we propose that P.kingianumvar.grandifolium should be recognised as a new synonym of *P.hunanense*. In addition, both morphology and phylogeny showed that *P.hunanense* is different from *P.kingianum* and has a close relationship with sect. Polygonatum.

### Taxonomic treatments

#### Polygonatum hunanense

Taxon classificationPlantaeAsparagalesAsparagaceae

H.H. Liu & B.Z. Wang

50E0C869-714C-59DE-B946-47EB803F1C55

=PolygonatumkingianumCollett & Hemsl.var.grandifolium D.M. Liu et W.Z. Zeng, Flora Sichuanica. 7: 230−231. 1981. Type: CHINA. Sichuan: Guan County, 900 m alt., 8 June 1978, *Hao Zhang 1231* (lectotype, designated here: CDCM [barcode 00044013]!, Figure 3; isolectotype: CDCM [barcode 00044022]!).

##### Modified description of *P.hunanense*.

Perennials, rhizome moniliform or ginger-like, 1−4 cm in diam. Stem straight or apex subscandent, 1−3.5 m. Leaves in whorls of 3−6 (–10), sometimes alternate or opposite near base and/or apex of stem, sessile, elliptic to oblong-lanceolate, 5–20 (–27) cm long, 0.5−5 cm wide, apex strongly cirrose or curved. Inflorescences (1−) 2−5 flowered; peduncle 1−4 cm long, pendulous; bracts at base of pedicel, subulate to lanceolate, 2−5 mm. Pedicel 0.4−1.8 cm. Perianth yellowish- or greenish-white, cylindrical campanulate, slightly constricted in the middle, 1.5−2.7 cm long, perianth segments 6, arranged into 2 whorls, each 3 lobes 4−6.5 mm. Stamens 6, filaments 2−3.5 mm long, stout and no thickening, anthers 2.5−5.5 mm long. Ovary superior, globose 4−7 mm in diameter. Style 8−14 mm long. Berries pale yellowish-green or orange, 1−1.8 cm in diam., 3−15 seeds.

##### Phenology.

It sprouts twice a year, in spring (March to April) and autumn (September). The spring-sprouting individual flowers from April to May and fruits from December to next February. The autumn-sprouting individual flowers from November to December.

##### Distribution and habitat.

*Polygonatumhunanense* is relatively common in Chongqing Municipality, Sichuan, Hubei and Hunan Provinces, China (Figure 1). It grows in evergreen broad-leaved forests, thickets or on moist grassy slopes, at an elevation of 200 m to 1200 m. In addition, it is also widely cultivated in those areas for harvesting the rhizomes.

##### Conservation status.

To our knowledge, this species is widely distributed in low elevations of southwest China. Therefore, we propose to treat it as Least Concern (LC) according to the IUCN Red List Categories and Criteria version 14 (August 2019). However, due to the medicinal value of the genus, many of its populations are destroyed by unmanaged exploitation.

##### Other specimens examined.

China. **Chongqing Municipality**: Nanchuan District, Sanquan, 24 January 1984, *Zhengyu Liu 4958* (fl., IMC!); *ibidem*, 10 July 1991, *Zhengyu Liu 917801* (fr. IMC!); Nanchuan District, Jinfo Mountain, 13 September 1985, *Zhengyu Liu 851732* (fr. IMC!); *ibidem*, 28 June 1999, *Zhengyu Liu 974488* (fl., IMC!); *ibidem*, 28 June 1999, *Zhengyu Liu 974498* (fl., IMC!); *ibidem*, 28 June 1999, *Zhengyu Liu 990498* (fl., IMC!); *ibidem*, 28 August 1999, *Zhengyu Liu 975059* (fr. IMC!); Jiangjin District, Simian Mountain, 16 July 2000, *Zhengyu Liu 2004036* (fl., IMC!); Pengshui County, Longmenxia, 24 June 1988, *Fading Fu & Yaling Cao 0264* (CDBI!); Qijiang District, Zhongfeng Town, Lianghekou, 22 October 2012, *The Qijiang team 13-500222-LY-411-01* (IMC!); *ibidem*, 22 October 2012, *The Qijiang team 13-500222-LY-411-02* (fr., IMC!); Qijiang District, Shihao & Wanlong, 12 October 2014, *The Qijiang team Qianjiang-0310* (IMC!); Wuxi County, Shuangyang, 16 July 1996, *Zhengyu Liu 760044* (fl., IMC!). **Hubei Province**: Enshi City, Hegongwei Village, 06 November 2016, *Jinping Si & Jingjing Liu 33-4* (HZU!). **Sichuan Province**: Pengzhou City, 22 June 1978, *Tianfu Yang & Yunjin Chen 1231* (CDCM!); Qionglai City, 11 June 1979, *Chengdu University of TCM 0668* (fl., CDCM!).

## Supplementary Material

XML Treatment for Polygonatum hunanense

## Supplementary materials

Supplementary material 1 Figure S1. Longitudinal section of flower of *P.hunanense* and *P.kingianum* Data type species data https://binary.pensoft.net/file/530894This dataset is made available under the Open Database License (http://opendatacommons.org/licenses/odbl/1.0/). The Open Database License (ODbL) is a license agreement intended to allow users to freely share, modify, and use this Dataset while maintaining this same freedom for others, provided that the original source and author(s) are credited.Maoqin Xia, Ying Liu, Jingjing Liu, Donghong Chen, Yan Shi, Zhicong Bai, Yu Xiao, Chen Peng, Jinping Si, Pan Li, Yingxiong Qiu
