# Supplementary figures and images for: A new synonym of Polygonatum in China, based on morphological and molecular evidence
Source: PhytoKeys. 2021 Apr 12;175:137–49. doi: 10.3897/phytokeys.175.63383 (PMC8390792; doi:10.3897/phytokeys.175.63383)

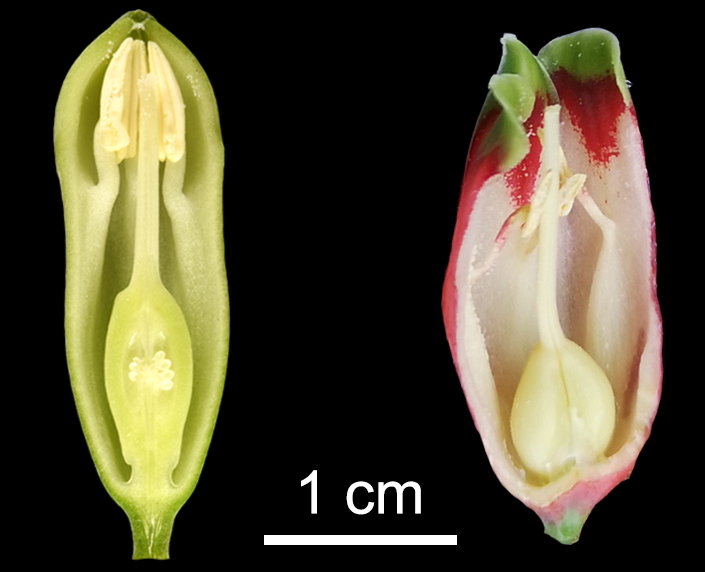

Supplement: Supplementary material 1 — Figure S1. Longitudinal section of flower of P.hunanense and P.kingianum [file phytokeys-175-137-s001.jpg]
